# SARS-CoV-2 and mitochondrial health: implications of lifestyle and ageing

**DOI:** 10.1186/s12979-020-00204-x

**Published:** 2020-11-09

**Authors:** Alistair V. W. Nunn, Geoffrey W. Guy, Wolfgang Brysch, Stanley W. Botchway, Wayne Frasch, Edward J. Calabrese, Jimmy D. Bell

**Affiliations:** 1 Department of Life Sciences, Research Centre for Optimal Health, University of Westminster, London, W1W 6UW UK; 2 The Guy Foundation, Dorset, UK; 3 MetrioPharm AG, Zurich, Switzerland; 4 UKRI, STFC, Central Laser Facility, & Department of Biological and Medical Sciences, Oxford Brookes University, Oxford, OX110QX UK; 5 School of Life Sciences, Arizona State University, Tempe, USA; 6 Environmental Health Sciences Division, School of Public Health and Health Sciences, University of Massachusetts, Amherst, MA USA

## Abstract

Infection with SARs-COV-2 displays increasing fatality with age and underlying co-morbidity, in particular, with markers of the metabolic syndrome and diabetes, which seems to be associated with a “cytokine storm” and an altered immune response. This suggests that a key contributory factor could be immunosenescence that is both age-related and lifestyle-induced. As the immune system itself is heavily reliant on mitochondrial function, then maintaining a healthy mitochondrial system may play a key role in resisting the virus, both directly, and indirectly by ensuring a good vaccine response. Furthermore, as viruses in general, and quite possibly this new virus, have also evolved to modulate immunometabolism and thus mitochondrial function to ensure their replication, this could further stress cellular bioenergetics. Unlike most sedentary modern humans, one of the natural hosts for the virus, the bat, has to “exercise” regularly to find food, which continually provides a powerful adaptive stimulus to maintain functional muscle and mitochondria. In effect the bat is exposed to regular hormetic stimuli, which could provide clues on how to resist this virus. In this paper we review the data that might support the idea that mitochondrial health, induced by a healthy lifestyle, could be a key factor in resisting the virus, and for those people who are perhaps not in optimal health, treatments that could support mitochondrial function might be pivotal to their long-term recovery.

## Introduction

The risk of severe morbidity associated with infection by SARS-CoV-2 rises with age and underlying co-morbidities, which indicate that up to 1.7 billion people, or 22% of the global population, could be at severe risk; the increased risk seems to be largely associated with an imbalanced and/or an excessive inflammatory response [1]. One suggestion is that the severity could be related to a failure of inflammation resolution, leading to pulmonary hyper-inflammation and “cytokine storms” [2]. With increasing age there is often an exaggerated innate immune response to respiratory infections [3] and rising inflammatory tone [4, 5]. Overall, it seems that susceptibility to the virus is related to an age-related loss of adaptive immunity combined with an increased innate immune response [6]. This “inflammaging” seems to be associated with T-cell immunosenescence and thymic atrophy; critically, exercise seems to be protective [7]. The protective effect of exercise is informative, as the pathological severity of SARS-CoV-2 infection seems to be associated with many obesity-related co-morbidities, such as diabetes [8–10], in contrast, physical fitness is emerging as a preventative strategy against the virus [11].

This suggests that as well as age, lifestyle could be important in determining susceptibility to the virus. We have suggested that a modern sedentary lifestyle has effectively removed exogenous hormetic stimuli, such as physical activity, which is leading to an accelerated ageing phenotype [12]. In short, a modern lifestyle could be accelerating the process of “inflammaging”: obesity is associated with a pro-inflammatory state, increased inflammatory macrophages and altered T-cell homeostasis [13]. In contrast, exercise is largely anti-inflammatory, which is thought to explain its many benefits [14, 15]. A key player in this adaptation is the mitochondrion, as mitochondrial stress enhances mitochondrial function not only in muscle, but in multiple other organs with myokines playing a key role [16, 17]. For example irisin, which protects mitochondria, can protect against ischaemia/reperfusion (IR) injury in the lung [18]. Irisin has also been found to favourably alter genes in adipocytes that are affected by the SARS-CoV-2 [19] and to modulate macrophage reactive oxygen species (ROS), displaying anti-oxidant and anti-inflammatory properties [20]. Critically, exercise can enhance mitochondrial function and capacity in peripheral blood mononuclear cells (PBMCs) [21].

As mitochondria are pivotal in the immune response and many viruses in turn modulate mitochondria [22, 23], it is possible that altered mitochondrial function may explain at least some of the variance in responses to SARS-CoV-2. As most cells in the body contain mitochondria, including immune cells, this would be expected and is now embraced by the concept of “immunometabolism”. This is perhaps most clearly seen in the clinical phenotype of subjects with inherited mitochondrial defects who often display immunodeficiency and a much higher rate of infections – highlighting the reliance of the immune system on mitochondria [24, 25]. Although this is relevant to resistance to the virus, it is also perhaps relevant to the efficacy of vaccines; Thacker and colleagues, using gene expression assays of PBMCs, have shown that there is an age-related decrease in response to influenza vaccines, which appears to be linked to decreased mitochondrial function [26]. In short, compromised mitochondrial function, either due to genetic factors, extreme age, or lifestyle, could have a bearing on both resistance to the virus and the ability to mount an effective response to a vaccine.

It therefore seems that maintaining “mitochondrial health” is vital, which probably correlates with an effective mitochondrial reserve induced by factors like physical activity, such that when the system is “stressed” (e.g., by a virus), it can cope. Although the virus may only infect certain cells, the immune response is global and dependent on mitochondrial function in multiple tissues and organs. What is clear is that severity is associated with the hyperinflammation syndrome and involves dysregulation of many different cell types [27]. This is to be expected, as throughout evolution, viruses have evolved to manipulate the immune system to hide from it, and can invoke immunosuppression, which in itself can become pathological, for instance, by modulating T-cells [28, 29].

It now seems that the spike protein of SARS-CoV-2 can bind to T-cell receptors (TCRs), acting as a super-antigen and causing excessive activation of the adaptive immune system – potentially resulting in the hyperinflammatory syndrome [30]. This is perhaps relevant as persistent antigenic stimulation can lead to T-cell exhaustion, which is associated with decreased oxidative phosphorylation and loss of mitochondrial function despite enhanced glycolysis – but can be reversed using anti-oxidants [31]. Data is now showing that COVID-19 patients do have populations of T-cells displaying mitochondrial dysfunction, as well as altered mitochondrial markers in monocytes – hinting that immune-metabolic phenotyping could be used to understand disease pathogenesis and possible treatments; this could include targeting mitochondria [32]. In short, the immune system itself could well be a target for this virus. Apart from the virus targeting the TCR as a super antigen, there is evidence that other than it binding to the angiotensin converting enzyme (ACE) as its main receptor, it may also bind receptors on immune cells, such as CD147 and CD26 [33], or neuropilin-1 (Nrp-1) [34, 35].

We have structured this paper to first review the now established data on general mitochondrial function and health in relation to “inflammaging”, followed by the evidence suggesting that the SARS-CoV-2 virus itself manipulates mitochondrial function and what we might learn from bats – which are thought to be its natural host. From this we propose that a poor lifestyle accelerates “inflammaging” which is associated with mitochondrial ill-health, and in some populations this predisposes them to a worse outcome. In the second part of the paper we discuss the implication of this idea in relation to current and suggested drug-based treatments and vaccine efficacy, the “long-COVID” syndrome, as well as how environmental factors may make some people more vulnerable. Understanding these concepts may help inform clinical strategy.

## Mitochondrial function in inflammaging and immunosenescence

Circulating extracellular vesicles (EVs) derived from immune cells seem to have emerged as a means of studying immunosenescence. In particular, they show an age-related decline in mitochondrial function – which could be related to dysfunctional mitophagy [36]. In fact, mice engineered to have dysfunctional T- cell mitochondria display accelerated senescence and “inflammaging”, highlighting the point that T-cells can determine organismal fitness and lifespan [37]. This does support data indicating the importance of a healthy T-cell response in defending against the virus [38, 39].

The underlying aetiology for “inflammaging” has long thought to be associated with mitochondrial dysfunction as suggested by Nick Lane in 2003 in his “double agent” theory [5], and is now receiving renewed interest, for instance, in how decreasing mitochondrial function can reduce T-cell function and enhance immune senescence, as mitochondria are pivotal in metabolic reprogramming towards the Warburg effect [40]. Indeed, as mitochondrial dysfunction can lead to “inflammaging”, the observed increase in older people of mitokines could be an attempt by the system to restore homeostasis as many are anti-inflammatory. Unfortunately, for many, this response doesn’t fully compensate [41]. This is why “exogenous” factors, such as physical activity or calorie restriction seem to be required to optimise function; these were normal factors during evolution, but are not in our modern sedentary and obesogenic environment.

One aspect of ageing is a failure to remove damaged components, for instance, dysfunctional mitochondria via mitophagy, which could lead to immune dysfunction [42]. It has been suggested that imbalances in mitochondrial mass could be responsible for ageing-related T-cell subset dysfunction [43], which would suggest a failure of mitophagy. Indeed, activation of mitophagy/autophagy is thought to be a pivotal mechanism in slowing ageing and inhibiting inflammation during calorie restriction (CR) [44]: CR/intermittent fasting has been suggested as a defence against the SARS-CoV-2 as it is anti-inflammatory [45]. In contrast, a modern sedentary lifestyle is also contributing to “inflammaging”, which acts as a common mechanism linking sarcopenia, obesity, cardiomyopathy and dysbiosis, with over-activation of nod-like receptor pyrin family domain containing 3 (NLRP3) inflammasomes and mitochondrial dysfunction playing key roles [46]. Overall, this all seems to support a close link between immunosenescence, inflammaging and failing mitochondrial function.

## Does SARS-CoV-2 modulate mitochondrial function, either indirectly or directly, and if so, in what cells?

The above suggests that there is a close link between mitochondrial dysfunction and immunosenesence, which could lead to an increased chance of an imbalanced immune response to SARS-CoV-2. This could take the form of both an inability to clear it, but also an exaggerated pro-inflammatory response and a “cytokine storm”. However there could also be another factor, and that is that the virus is modulating mitochondrial function to help it replicate.

One clue to this possibility is that many viruses do appear to manipulate bioenergetics towards aerobic glycolysis (the “Warburg effect”); this is a highly energy-dependent process to help generate substrates to build new virus particles [47]. Aerobic glycolysis does require healthy mitochondria, and is a normal process in multiple cell types, including immune cells [48]. Perhaps tellingly, data suggest that successful clonal expansion of vaccine-elicited T-cells is heavily depending on mitochondrial function [49]. What this suggests is that any cell forced to produce new viruses, if its mitochondria are not functioning optimally, could rapidly become energy deficient and be more likely to die, and depending on its type and location, could either enhance inflammation and/or compromise the immune response.

### What are the SARS-CoV-2 receptors and where are they found?

The direct impact of the virus will depend on which cells it infects. To date, most evidence points towards ACE2 being the primary receptor for this virus. Early data suggested ACE2 is predominantly expressed in pulmonary alveolar type 2 progenitor (AT2) and respiratory epithelial cells, but is also expressed in myocardial, illium and oesophagus, as well as some kidney cells – with little expression in immune cells [50]. Elevated ACE2 expression has also been found in the olfactory neuroepithelium, potentially explaining the anosmia that some patients have suffered [51]. More recent data has suggested that ACE2 may be primarily expressed in bronchial transient secretory cells [52].

Perhaps of relevance to the increased risk associated with obesity is that high ACE2 expression has been found in both visceral and subcutaneous adipose tissue; this is important as adipose tissue in obesity is well known to secrete higher levels of angiotensin 2, an inflammatory component of the renin-angiotensin aldosterone system (RAAS), which is key in driving many of the pathological complications associated with this condition [53]. Critically, obesity also seems to be associated with increased expression of ACE2 in the lung, and enhanced inflammatory markers and dysregulated lipogenesis; viruses are well known to hijack lipid metabolism as part of their life cycle [54].

Although ACE2 is not highly expressed in immune cells, it is possible that other proteins expressed on immune cells could be acting as SARS-CoV-2 receptors, such as CD26 (also known as dipeptidyl peptidase 4, DPP4) or CD147 (also called basigin). CD147 can be activated by cyclophilins, which are inhibited by cyclosporine A. Critically, the expression of these potential receptors changes with age, as well as with co-morbid conditions, such as obesity and hypertension [33]. Thus both CD147 and cyclophilin A have been suggested as potential targets for treating the virus. For example, cyclosporine is very effective against corona viruses; however, its immunosuppressive actions would limit its usefulness [55].

CD147 and ACE2 expression is often increased in lung disease, resulting in excessive activation of the RAAS and enhancing damage, which could, in part, explain the origins of the cytokine storm. It has been suggested that melatonin, a potent natural anti-oxidant, could suppress the CD147 inflammatory pathway and help in treating COVID-19 patients [56]. In silico binding studies do seem to support the possibility that the virus does indeed use CD147 as a receptor, and could, potentially, explain why lymphopenia is associated with severity of COVID-19 and a loss of T-cell subsets [57].

Data is indicating that this virus may also bind to neuropilin-1 (Nrp-1); this protein is expressed on many cells, including those in the central nervous and immune systems, and is also a receptor for vascular endothelial growth factor A (VEGF-A) [34, 35, 58]. Apart from suggesting it can thus potentially infect the central nervous system (CNS), it also appears that SARS-CoV-2 can induce analgesia – which could aid in increased disease transmission in asymptomatic individuals [59]. Nrp-1 is also a focus for immunotherapy treatments in oncology, as it is expressed on subsets of regulatory T-cells [60]. There is also data indicating it is expressed in the cardiovascular system; if its expression is reduced, it results in cardiac mitochondrial dysfunction as it controls the master mitochondrial regulator, peroxisomal proliferator-activated receptor γ coactivator 1α (PGC1α), as well as peroxisomal proliferating activating receptor γ (PPARγ) [61].

This data does suggest that the virus not only modulates essential components of the RAAS affecting inflammatory balance via ACE2, but if it is also modulating the T-cell response directly, for instance, via CD147, or the TCR, or even, Nrp-1.

### Do SARS viruses code for proteins that target mitochondria?

In SARS-CoV-1 the open reading frame-9b (ORF-9b) encodes for a protein that locates to the mitochondrion. Here it induces fusion by triggering degradation of dynamin-like protein 1 (DRP-1), while inhibiting mitochondrial anti-viral signalling proteins (MAVS). This is thought to underlie its ability to suppress the anti-viral interferon response. It can also induce autophagy and activate NF-κB [62]. MAVS are small proteins that on detection of double stranded RNA (dsRNA) oligomerise on mitochondria to form a signalling platform and initiate interferon signalling, as well as cell death [63, 64]. It also seems that MAVS can act as adaptor proteins for NLRP3, forming a complex with mitochondria, although the inflammasome can also be activated in a way that doesn’t induce an interferon response, but can induce the interleukin beta (IL-β) response [65].

With regards SARS-CoV-2, protein interaction mapping shows that it shares a great deal of homology with SARS-CoV-1, but significantly, several of its proteins are also predicted to directly interact with mitochondria, such as non-structural proteins (NSPs) 4 and 8, and ORF9c, as well as components of the interferon and NF-κB pathways [66]. This, because of the well described role of viruses in manipulating mitochondrial function, has led to other groups suggesting that indeed, mitochondrial “hijacking” by SARS-CoV-2 could be a key factor in the pathogenesis of this virus [67].

Many viruses also use viroporin proteins that can oligomerise to help viral entry and release, as well as control intracellular signalling ions, such as calcium or potassium. They can also, via direct protein interaction, manipulate signalling pathways. The host cell detects these as changes in ions levels and ROS, and via, for instance, the NLRP3 inflammasome, activates cellular defence [68]. SARS-CoV-1 has at least three viroporins, two of which are essential for replication and virulence [69]; the E protein, in particular, not only seems to trigger P38 MAPK activity, but also seems to modulate calcium flux by acting as a permeable ion channel in endoplasmic reticulum-Golgi intermediate compartment (ERGIC)/Golgi membranes, activating the inflammasome [70]. SARS-CoV-2 seems to have a similar E viroporin that induces ionic imbalance [71]. From the calcium and ROS signalling perspective this is particularly important, as mitochondria are not only pivotal in calcium buffering and signalling, but are also controlled by calcium [72]. Data suggest that many viruses form viral “factories”, which are constructed from host cell membranes, and are often tightly coupled to mitochondria to provide precursors and energy – this includes the *Coranoviridae* [73, 74].

### SARS-Cov-2 may enhance aerobic glycolysis to favour replication

Emerging data is now suggesting that T-cell mediated immunity may be playing a powerful role in protecting against the virus, as many asymptomatic people, or those who have only had mild symptoms, show low levels of anti-SARS-CoV-2 antibodies but a strong T-cell mediated response against the virus. In contrast, more severe disease is associated with more rapid seroconversion and the presence of inflammatory markers, such as C-reactive peptide (CRP) [75, 76]. In fact, it now appears that the severity of infection positively correlates with a decreased type 1 interferon (IFN1) response, but an exaggerated inflammatory response, characterised by high levels of interleukin 6 (IL-6) and tumour necrosis factor alpha (TNFα) – possibly related to excessive activity of nuclear factor kappa B (NF-κB). This latter finding could be related to an auto-inflammatory loop in the lungs [77]. It does seem that in some people that the transcriptional response to SARS-CoV-2 is imbalanced, with a less than optimal interferon-I and -III response, but an exaggerated chemokine one; this may represent an evolved manipulation of the immune system by the virus that worsens the outcomes for older patients with co-morbidities as they cannot clear the virus properly [78].

Data from autopsies of deceased COVID-19 patients show that tissue inflammation and organ dysfunction do not map to the cellular distribution of the virus, hinting at tissue-specific tolerance. In fact, severe inflammatory changes seem to be largely restricted to the lungs and the reticulo-endothelial system. This suggested that COVID-19 related deaths were due to immune-mediated, rather than pathogen-mediated organ inflammation and injury [79]. It may therefore be relevant that IFN1 can also have some anti-inflammatory actions, modulating for instance, NLRP1/3 inflammasomes and inhibiting interleukin-1 (IL-1) production [80]. Type 1 interferons are key in modulating T-cell responses and resistance to viruses [81, 82].

It had been suggested that as the virus uses ACE2 as a receptor on the cell surface it could trigger activation of the renin-angiotensin-aldosterone system (RAAS), which in turn, leads to hyperactivation of the NLRP3 inflammasome and pyroptosis, a form of cell death that results in inflammatory amplification [81]. Data does now seem to support this and has been shown in various types of human stem cells – which could potentially affect tissue regeneration [83]. ACE2 cleaves angiotensin II to generate angiotensin (1–7), which is largely anti-inflammatory and protective [84]. Critically, mitochondria have a functional angiotensin system [85], and ACE2 seems to be mitochondrially protective [86]. Potentially of interest here is that a product of ACE2, angiotensin-(1–9), seems to inhibit mitochondrial fission in the heart, enhancing mitochondrial fusion and calcium buffering and protecting against cardiac hypertrophy [87]. It is thus possible, by binding to ACE2, the virus may suppress a counter-balancing anti-inflammatory pathway that affects mitochondrial function.

So why would SARS-CoV-2 do this? One possible explanation is that the virus affects the most prevalent immune cells in the lungs, monocytes/macrophages, inducing them to shift metabolically to aerobic glycolysis, which favours viral growth. The infection, in the presence of oxygen, seems to achieve this by triggering mitochondrial reactive oxygen species (ROS) production, stabilising the hypoxia-inducible factor-1α (HIF-1α), which in monocytes, consequently inhibits T-cell responses and lung epithelial cell death. It seems that high glucose levels induce viral replication [88]. Furthermore, the inflammasome can also modulate glycolysis; in macrophages, this may be a key process in metabolic reprogramming [89]. Critically, inflammasome activation can be inhibited by nuclear factor, erythroid 2-like 2 (Nfe2l2/Nrf2), which is pivotal in enhancing antioxidant defences and suppressing inflammation [90]; it therefore counterbalances NF-κB, which is also redox activated, but central to the immune response [91].

Another key factor is that the SARS-CoV-2 genome encodes proteins that can target the NF-κB pathway [66]. SARS-COV-2 therefore seems to induce a Warburg shift (aerobic glycolysis), which is a tactic that many other viruses, and cancer cells, use [47]. It is thus of relevance that that the metabolic reprogramming induced by SARS-CoV-2 can be suppressed by melatonin [92], which is a powerful antioxidant that protects mitochondria [93]. In fact SARS-CoV-2 also seems to induce activation of pathways like p38 mitogen activated protein kinase (MAPK), which results in cell cycle arrest, inhibition of apoptosis, and results in a feed-forward inflammatory loop [94]; the systems it targets therefore do seem have much in common with those that are altered in cancer [95]. Critically, MAPKs also modulate mitochondrial function, for instance, interacting with the voltage dependent anion channel 1 (VDAC1) [96]. This seems to add up to the virus manipulating several pathways to invoke aerobic glycolysis, which must involve mitochondrial function.

Diabetes is also associated with activation of p38 MAPK via ROS generated by glucose induced mitochondrial dysfunction that can be offset by targeted mitochondrial antioxidants [97, 98]. Not only is diabetes a risk factor for a worse outcome when infected with SARS-CoV-2, but the virus itself may induce a worsening of the condition [99–101]. Indeed, it now seems that fasting blood glucose is a predictor of mortality for COVID-19 patients [102]. Overall, prediabetes and/or type 2 diabetes (T2D) itself is embraced by the concept of the metabolic syndrome in which insulin resistance, mitochondrial dysfunction and inflammation are all components [12]. Metformin, which modulates mitochondrial function, is a key treatment for T2D [103] – and has shown some benefit in COVID-19 patients [104, 105]. In contrast, evidence indicates that the inflammatory effect of the Western diet may induce activation of the NLRP3 inflammasome [106]. In light of the emerging data, this could only worsen the potential for an exaggerated inflammatory response.

### SARS-CoV-2 could lead to mitochondrial stress

**Fig. 1:**
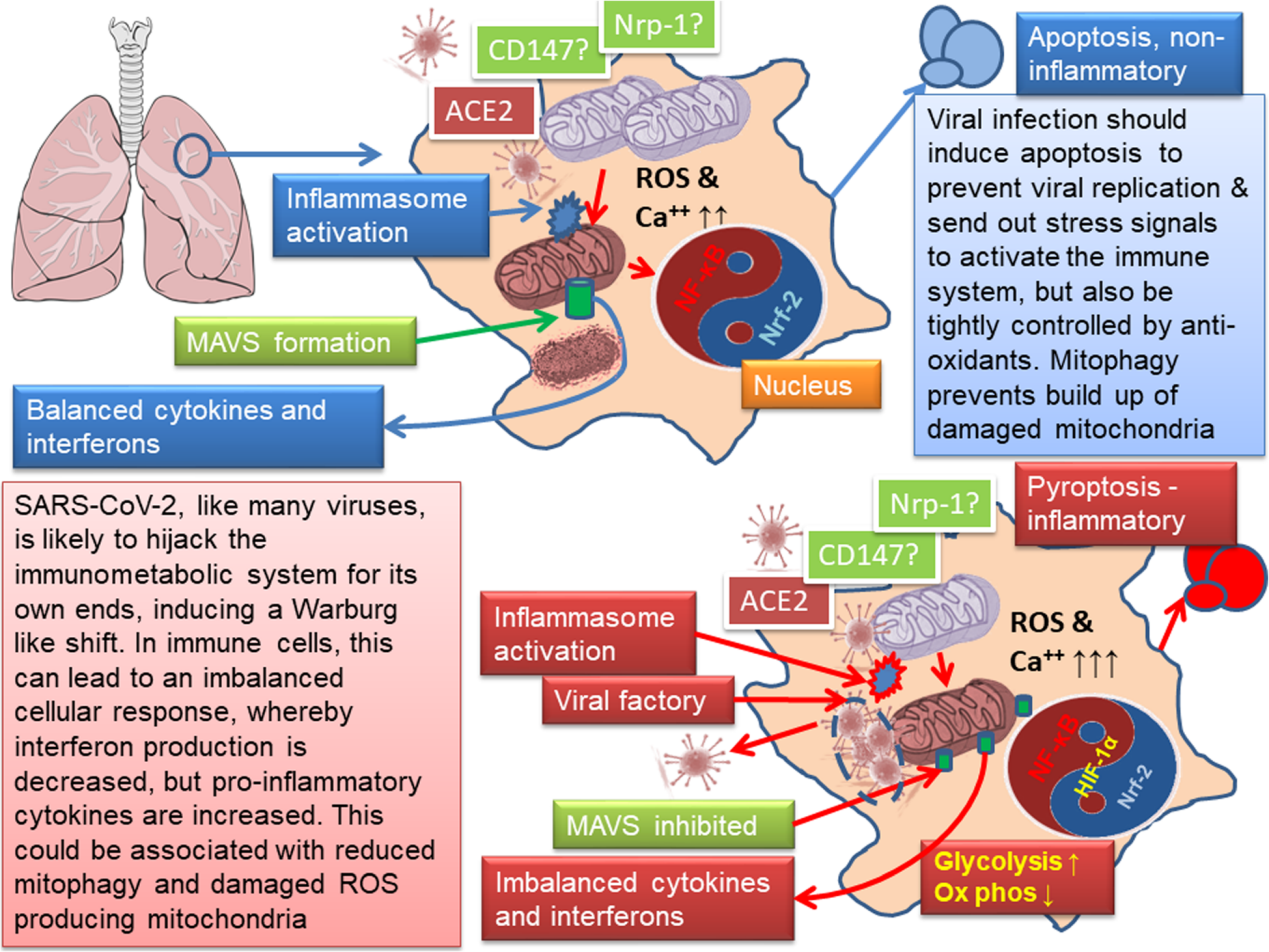
Viruses like SAR-CoV-2 manipulate cellular metabolism leading to the potential for a feed-forward inflammatory loop. Viruses have evolved to usurp their host’s cellular machinery to make more viruses. One common mechanism is to suppress apoptosis and manipulate the immune system to inhibit specific anti-viral programmes, which usually means interferons, while stimulating a shift towards aerobic glycolysis to provide precursors to build new viruses. However, this latter ability repurposes pathways that are often involved in generalised immunity that both increase the production of pro-inflammatory mediators, while metabolically reprogramming immune cells. In the case of SARS-CoV-2 this may well result in a feed-forward pro-inflammatory loop in the lungs, which seems to be driven by monocytes/macrophages switching to aerobic glycolysis and is driven my mitochondrial ROS and stabilisation of HIF-1α; in turn, this metabolic shift suppresses T-cells and the interferon response [88]. This process is accentuated as the virus may well stimulate inflammasome activation [81], while if it is similar SARS-CoV-1, it could also suppress MAVS formation and activate NF-kB [62]; protein interaction mapping does suggest this is the case [66]. As it is likely that inflammasome activation can also invoke glycolysis [89], then the evolutionary rationale seems sound. Of particular importance here is also the balance between NF-kB and Nrf2, which more or less seem to counter-balance each other, as Nrf2 is pivotal in suppressing excessive oxidative stress [91]. For more detailed reviews of the role of mitochondria in the immune response see [22, 107]

It is therefore likely that SARS-CoV-2 does modulate mitochondrial function. So it could be surmised, for instance, that this virus could ensure close tethering of mitochondria, and via calcium flux, stimulate their function. Clearly, if this process was too overwhelming, or the mitochondria were already functionally compromised, this would rapidly lead to mitochondrial stress. With regards this, Singh and colleagues have highlighted an interesting link with viruses and the production of mitochondrially-derived vesicles (MDVs), which are normally part of a system to remove damaged components from the mitochondrion [67]. If, like SARS-CoV-1, this new virus also does this, and also induces mitochondrial fusion, it hints at an interesting ability to prevent apoptosis, as well as mitophagy, but stimulate a mechanism to move virus particles around. If it is also inhibiting MAV activity, then the mitochondrion might not initiate interferon signalling, but might still continue, potentially by producing higher than normal levels of ROS, to stimulate inflammasome activity and metabolic reprogramming towards glycolysis. In effect, the virus repurposes the normal inflammatory metabolic reprogramming towards aerobic glycolysis, which involves modulation of mitochondrial function, but manages to suppress the normal anti-viral interferon response. In many tissues, the system may manage to stay in balance and not cause an overt over activation of the immune system, but in the lungs, it seems that in some people, this balance is lost. Figure 1 summarises this.

## The immune system, hormesis and mitochondria

As indicated, if the virus is modulating mitochondrial function in a variety of cell types, either directly, or indirectly, then the more robust the mitochondrial system, the greater the chance of the system being able to resist the virus. In general, hormetic factors, such as exercise, seem to be necessary to maintain mitochondrial health throughout the body; this phenotype is associated with a more balanced immune response and minimisation of “inflammaging”. In this section we review why this is, and look at why one of the natural hosts of the virus, the bat, may be able to resist.

### A robust mitochondrial system and effective immune system may rely on hormesis

A key component of effective immunity is now thought to be a healthy mitochondrial system [107], while an underlying unifying element to both the ageing process and conditions associated with a poor lifestyle is a degradation in overall mitochondrial function/reserve and a rise in oxidative stress and inflammation [4, 5]. An important factor in the maintenance of mitochondrial function is hormesis where low levels of stress induce an over-compensatory response that induces positive adaptations, enabling an organism to better tolerate the stressor next time they encounter it. For example, an effective hormetic response can be induced by sub-lethal doses of physical activity, calorie restriction and many plant polyphenols [12], with mitochondrial stress being a key trigger [108]. This results in an enhanced respiratory reserve and anti-oxidant capacity, and a greater ability to manage the ATP/ROS ratio when placed under stress [109]. Certainly, small, long-lived species like bats and sparrows, when compared to comparatively much shorter lived species like mice, do demonstrate lower levels of mitochondrial hydrogen peroxide release [110]. Given that mitochondrial dysfunction is strongly correlated to immune dysfunction and chronic inflammation [111], then inflammation resolution is probably going to be best achieved by ensuring healthy mitochondrial function as it ensures that ROS release does not get out of control.

### What can bats tell us?

The concept of hormesis suggests that it is important to constantly stimulate the renewal and maintenance of a large population of healthy mitochondria. It may therefore be possible to learn something from one of the natural hosts of SARS-CoV-2, bats [112]. Bats are the only true flying mammal and are exceptionally long-lived for their size. This could be because the evolution of flight has required a whole host of adaptations, including maintaining a large pool of mitochondria that produce very little ROS while maintaining a high ATP output. This appears to have gone hand-in-hand with changes in the immune system to prevent excessive inflammatory activation by stressed mitochondria, for instance, by dampening NLRP3 Inflammasome activity. The net result is that many bats can tolerate high levels of viruses, like the *Coronaviridae* family [113–116] and do show a reduced antibody and inflammatory response, hinting they are using another part of their immune system to control the virus [117].

The inflammasome may thus be important, as its activation can lead to pyroptosis, an inflammatory form of apoptosis, and can be triggered by excessive mitochondrial stress [118]. It may well be an essential component in “inflammaging” [42]. There is some evidence that at least in some species of bat, mitochondrial health, despite bursts of oxidative stress, is maintained by stringent mitochondrial quality control mechanisms, like mitophagy [119]. Mitophagy is in fact a negative regulator of NLRP3 inflammasome activity, so although mitochondrial damage can activate the inflammasome, it can also activate counter-balancing mitophagy to prevent excessive inflammation [120]. In short, it seems that powered flight has required the co-evolution of both mitochondria that tightly control ROS, and a co-adapted immune system.

Critically, there is evidence that SARS-CoV-2 inhibits autophagy [121], suggesting it might also inhibit mitophagy. If this virus does indeed induce mitochondrial fusion, as SARS-CoV-1 may do [62], then this would fit, as mitochondrial fusion can inhibit mitophagy, and can inhibit cell death and ensure energy production, although prolonged fusion can also initiate cell death in some circumstances [122]. This latter point suggests another innate anti-viral mechanism. Overall, modulation of the inflammasome could be one element in how the virus could result in an “inflammaging” phenotype.

### Humans, hormesis, exercise and the immune system

The effects of hormesis, certainly for humans, are perhaps most clearly seen in response to exercise training, in particular, aerobic training, where both mitochondrial capacity and function is increased in young and old [123, 124]. This is matched by increased survival and healthier ageing in cohorts who undertake plenty of physical activity [125]. Active muscle is generally inflammatory, but commensurately induces counterbalancing powerful anti-inflammatory and anti-oxidant mechanisms throughout the body. Exercise thus appears to show a biphasic dose response and the evidence is building that as long as it is not done excessively, in particular, allowing time for recovery, it is highly beneficial: over time the adaptive over-compensation includes an improved anti-inflammatory and anti-oxidant feedback (25-28) [126].

Muscle has now been shown to have other functions, like harbouring and supplying anti-viral stem T-cells, hence, antagonising T-cell exhaustion and protecting proliferative potential during inflammation [127]. In contrast white adipose tissue plays a key role in adaptive immunity, and in excess, contributes to the altered immune function and chronic inflammation often associated with obesity [128]. In particular, excessive visceral adipose tissue (VAT), seems to play a pivotal role in obesity-related pathogenesis; critically, its volume is decreased by exercise [129]. Furthermore, not only does type 1 interferon unlock dormant adipocyte inflammatory potential [130], but exercise reduces adipose expression of NLRP3 [131]. It therefore seems that adipose tissue and muscle play a yin-yang role in the immune response, whose set point will thus be determined by an individual’s fitness and calorie balance, and overall mitochondrial capacity and health, and thus, reserve. In short, mitochondrial reserve, and thus spare respiratory capacity, is pivotal in enhancing the “healthspan”, and is greatly improved by exercise [109]. The key here is that stress can be signalled from mitochondria in any tissue to the rest of the body by way of “mitokines”; muscle activity is a prime inducer of mitochondrial stress [132].

### Mitochondrial reserve and redox

It therefore seems that control of inflammation is associated with tight control of mitochondrial ROS, which is itself dependent on “mitohormesis” by factors such as exercise, plant compounds in the diet, and calorie restriction [108, 133]. The basis for this is that life is based on redox and compartmentalised production of ROS as part of a signalling system [134, 135]. This has led to redox theories of disease and ageing, focussing on the mitochondrion [136] and their role in generating an age-related rise in inflammatory tone [5], which supports the pivotal role of mitochondria in the immune system [137] and in resistance to infections including viruses [138]. In support of this, there is increasing evidence that mitochondria can also act as *net sinks* of ROS and this is linked to lifespan. For instance mitochondria from the long lived naked mole rat (NMR) produce less ROS than comparable shorter lived animals [139, 140]. Furthermore the mitochondria in NMR, and bats, also appear to be able to maintain a depolarisation of the inner membrane for much longer during their life cycle, which is a key mechanism to reduce ROS production during ageing [141]. A key idea that relates to this is the Redox-Optimised ROS Balance (R-ORB) hypothesis, which stipulates that mitochondrial emission of ROS will reach a nadir when respiratory rate reaches a maximum – in effect, mitochondria will maximise ATP production and minimise ROS as they evolved to work at an intermediate redox state [142, 143]. Thus having a good mitochondrial reserve might suggest that this nadir can be maintained when the system is put under stress.

An essential component of mitochondrial control is uncoupling. This is a process whereby the proton gradient in the mitochondrion is uncoupled from ATP production, and it initially seemed to be a key process to reduce ROS production, as well as generating heat. It was therefore thought to act as a very good safety valve for mitochondria and play a fundamental role in survival and prevention of oxidative damage. In fact, 20% or more of the energy captured by electron transport is dissipated. However, uncoupling can also be associated with an increase in ROS, hence, it is a key component of redox signalling – and has led to updated versions of the “uncoupling to survive” hypothesis. It may therefore play a key role in mitohormesis, resulting not just in cell autonomous adaptations, but also systemic adaptation from signals, for instance, sent out from stressed skeletal muscle via mitokines. Uncoupling also controls calcium signalling. It now seems that mild uncoupling can, indeed, lead to increased longevity [144]. It is thus perhaps relevant that a mitochondrial uncoupling protein, UCP2, can negatively control the inflammasome [145], and in general, seems to suppress immune activity [146].

Uncoupling thus plays an important role in mitochondrial efficiency, which can either be defined as the respiratory control ratio (RCR - ratio of mitochondrial respiration supporting ATP synthesis to that required to offset the proton leak) or the ATP/oxygen ratio (the amount of ATP generated per unit of oxygen consumed) – this can lead to some confusion, as it can lead to opposite conclusions about efficiency. However, whichever metric is used, it does describe the capacity to convert resources into ATP, and in effect, the coupling efficiency [147]. In fact a study has shown that skeletal muscle mitochondria in obese, sedentary and insulin resistance women somewhat paradoxically show reduced mitochondrial coupling, but a higher production of mitochondrial hydrogen peroxide. In effect, despite a degree of uncoupling, their mitochondria were showing signs of oxidative stress; this might have been due to nutrient overload. However, an exercise training programme corrected this, and was correlated with an improvement of mitochondrial function, in particular, an enhanced ability to undertake beta oxidation of fats and restoration of metabolic flexibility, the ability to switch between carbohydrate and fat as energy sources, and better insulin sensitivity [148]. The apparent increase in uncoupling could be part of a homeostatic response to reduce excessive ROS production, as UCPs can be activated by oxidative stress [149]. This would further support evidence that exercise induces an adaptive response that enabled the mitochondrial system to cope better.

Finally, it is perhaps worth emphasising the link between mitochondrial reserve and ability to control oxidative stress. Mitochondria can generate ROS and are closely linked to Nrf2, which is a master transcription factor controlling antioxidant responses [150]. This suggests that exercise will not only induce greater mitochondrial reserve, but greater anti-oxidant capacity – and perhaps, a greater reserve ability to uncouple to manage oxidative stress.

### Ageing, immune system reserve and immunosenescence

As previously indicated, like the original SARS virus, this new virus also seems to induce worse outcomes in patients who are older, have hypertension and cardiovascular disease, and induces a phenotype characterised by raised inflammatory and coagulation markers, multi-organ failure, as well as neurological complications and myocardial injury [151]. In short, most things we identify with the ageing process and the metabolic syndrome, both of which are associated with declining mitochondrial function [152, 153]. It thus pertinent that the rate of ageing can be modified by lifestyle and disease, and that epigenetics are making it possible to determine, with some degree of accuracy, the biological age and compare it with the chronological age through DNA methylation (DNAmAge), and predict the likelihood of future mortality [154, 155]. Although mitochondria obviously play a role in this, and reduced mitochondrial DNA copy number (mtDNAcn) does appear to be a proxy for mitochondrial buffering capacity, and is negatively correlated with DNAmAge, the precise relationship with biological age is still unclear. For instance, evidence does indicate a clear role for mitochondria in ageing-related disease and mortality, but not necessarily chronological age [156]. However, data does suggest that inducing mitochondrial dysfunction alone in T-cells can induce premature senescence, driving “inflammaging” and a tendency towards a cytokine storm [37]. One well known concept in ageing is the idea of declining organ reserve, which at the molecular level, is related to a loss of excess metabolic capacity – in particular, bioenergetics and mtDNA, as well excess telomere capacity [157]. In this respect, it could be argued that the immune system could be viewed as an organ, and is also subject to declining reserve.

As the immune system ages there is a subclinical accumulation of pro-inflammatory factors, as well as decreased numbers of circulating respiring mitochondria found in extracellular vesicles (EVs), which are derived from immune cells [36]. Coupled to this, there is also evidence that with increasing age the monocyte inflammasome-mediated inflammatory response is altered. For instance, this response to influenza A is retained but the anti-viral interferon response declines [158]. Furthermore, ageing is also associated with a gradual loss of anti-oxidant capability that is associated with a decrease in the T helper 1 (Th1) anti-viral response, which might underlie some of the anti-viral activity of glutathione and other anti-oxidants [159, 160]. This is certainly commensurate with reduced immune system reserve.

However, there is still a lot that is not understood about ageing, which is why it has led some authors to categorise it using several separate hallmarks, with mitochondrial function only being one of several integrated systems as the precise cause is still not fully understood [161]. However, many authors continue to focus on the mitochondrion – mainly because it represents an ancient nexus that arose from the endosymbiotic event between a prokaryote and Archaean that gave rise to eukaryotes, and understanding this does provide insight into the immune system and inflammation, and the ageing process [152, 162]. Some have suggested that ageing is actually related to the loss of mitochondrial respiratory reserve capacity [109]. This, we suggest, does have a great deal of merit, and in relation to resistance to viruses, could be viewed from a reduction in bioenergetic/redox capacity of the immune system as people age. Tellingly, reduced skeletal muscle mtDNAcn is associated with symptoms of the metabolic syndrome, whereas exercise increases mtDNAcn and is negatively associated with markers of the metabolic syndrome and enhanced aerobic capacity [163]. Thus although certainly not the entire story, ageing is associated with declining mitochondrial function, which is likely to be related to reduced immune “reserve” and flexibility. Hence, as a proxy for potential severity when infected, mitochondrial function does have its place in the ageing process.

**Fig. 2:**
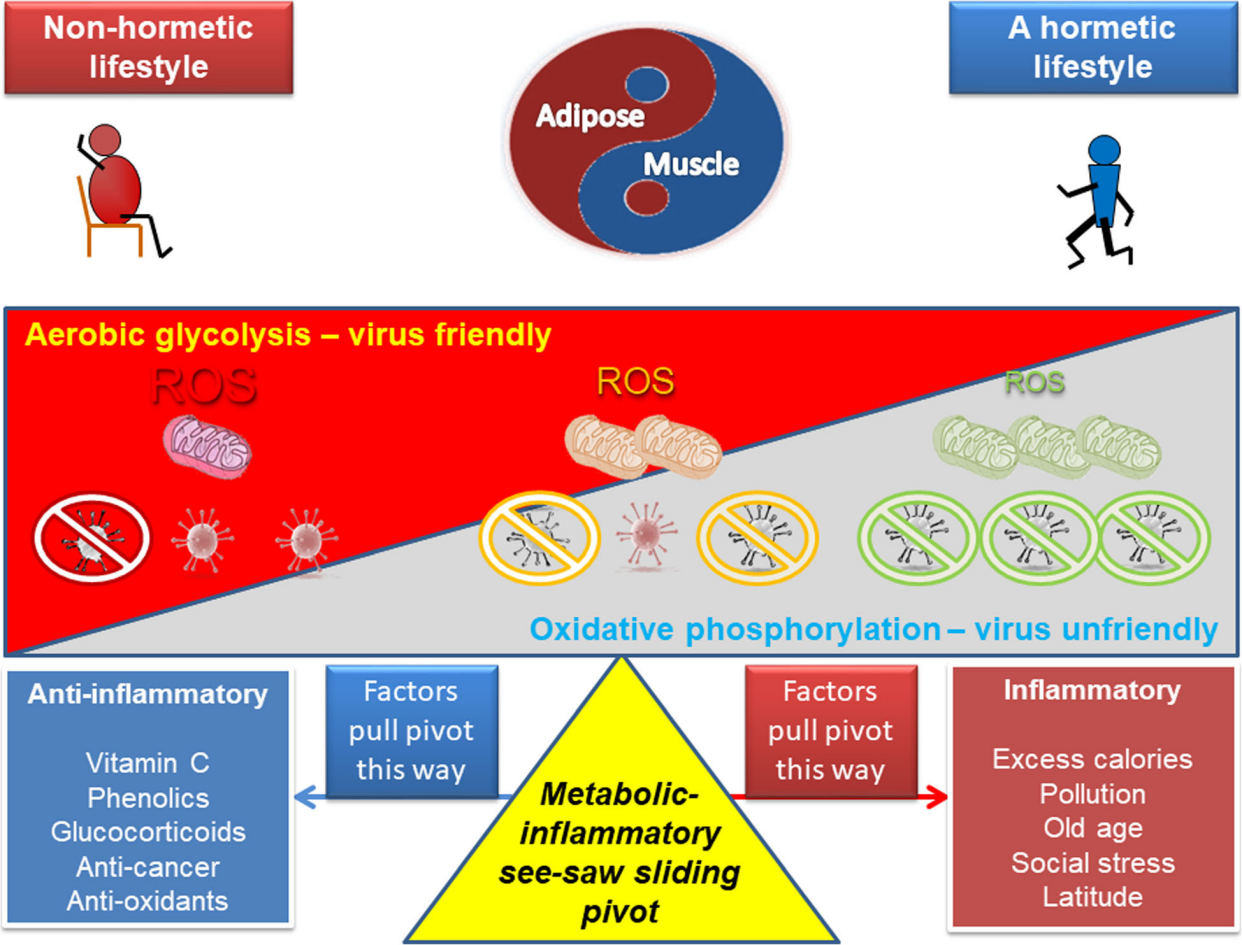
Resistance to SARS-CoV-2: mitochondrial and redox reserve, hormesis and the metabolic/inflammatory see-saw. Data strongly support that people who are aerobically fit, and follow a healthy lifestyle, tend to have a greater metabolic and anti-oxidant reserve related to training-induced mitochondrial adaptation; this is associated with less disease and a longer “healthspan”. The underlying principle that leads to this is described by hormesis and is the product of humans having evolved, along with most other animals, in an environment where this adaptation was constantly induced due to the need to move, and food was less available. As a phenotype, they tend to have greater muscle to fat ratio, and exhibit few, if any symptoms of the metabolic syndrome, such as increased liver fat and visceral adipose tissue (VAT) volume, insulin resistance, or markers of chronic inflammation. In this context, data suggest that muscle, as an organ, tends to be anti-inflammatory when used regularly, whereas adipose, in particular, in VAT is inflammatory if it becomes overloaded. As the virus seems to induce oxidative stress [164] and aerobic glycolysis to enhance its own replication, it could be argued that in a person with poor mitochondrial and anti-oxidant reserve, infection will tend to tip towards chronic inflammation and incomplete resolution. In this scenario, it is important to suppress either inflammation itself, or provide extra anti-oxidant power to damp down the potential for a feed-forward inflammatory spiral

The lessons for humans are thus fairly clear: exercise is part of our evolutionary heritage, and plays key role in maintaining optimal mitochondrial health and immune balance (Fig. 2).

## Mitochondrial function and therapeutic strategy

As is becoming clear, maintaining good health, in particular, optimal levels of aerobic fitness and muscle/fat balance is a good preventative strategy, in effect, the results of living a healthy lifestyle. A retrospective study following the Hong Kong influenza outbreak in 2008 found that physical activity was protective and displayed a “U” shaped dose-response curve [165]. High aerobic fitness is associated with reduced morbidity and mortality [166, 167] and physical activity, if not overdone, is generally anti-inflammatory in the longer term [15, 168] and results in an enhanced anti-oxidant status [169]. Equally, calorie restriction, which is associated with improved lifespan, is also anti-inflammatory [170, 171], as is a diet high in polyphenols and probiotics [172].

However, there are still many people, who, for various reasons are not living a particularly healthy lifestyle – the effects of which become worse with age. This therefore raises the question, would enhancing/supporting mitochondrial health help in this population if they become infected and would understanding the role of mitochondria help in deciding treatment regimens? There are a number of possibilities, ranging from suppressing the auto-inflammatory loop (e.g., direct targeting of inflammatory pathways, with, say antibodies, or kinase inhibitors), to mitochondrial protection, enhancing mitochondrial turnover and renewal and preconditioning, to direct management of redox. In fact, it seems that many established drugs probably already do modulate mitochondrial function, which may provide us with further insight.

The other main strategy and one perhaps with the greatest potential preventative benefit in the long run, is vaccination. The implications of mitochondrial health here could be extremely important in whether or not a vaccine is successful in particular populations, for instance the elderly and those with co-morbidities.

### Repurposing drugs

Many of the compounds now being studied may influence mitochondrial function. For instance research on immunomodulation during influenza infections has looked at corticosteroids, peroxisomal proliferating activated receptors (PPAR) agonists, cyclooxygenase (COX) inhibitors, adenosine monophosphate kinase (AMPK) activators, direct antioxidants, and natural products [173]. All of these can modulate mitochondrial function [174–178]. Non-steroidal anti-inflammatory drugs (NSAIDs) in general, to varying degrees, affect mitochondrial function [179]. Critically, network-based drug repurposing has recently identified several candidates, such as irbesartan, paroxetine, sirolimus, melatonin and quinacrine, amongst others [180]. It has been suggested that angiotensin receptor blockers (ARBs) are mitochondrially protective [181], while anti-depressants, such as paroxetine, can inhibit mitochondrial function [182]. Sirolimus, or rapamycin, is actually one of the best studied calorie restriction mimetics as it modulates mammalian target of rapamycin (mTOR); it is anti-inflammatory and modulates mitochondrial function, and could play a key role in mitohormesis [183]. It can, in fact, increase mitochondrial respiration and reduce production of hydrogen peroxide [184]. Critically, data does suggest that this new virus can indeed inhibit autophagy and the mTOR pathway [121]. This might suggest that it can enhance ATP production while reducing ROS, which would obviously benefit it (by at least, initially, suppressing immune activation). Overall, it could be argued that compounds that do inhibit mitochondrial function might have a number of effects, such as inducing mitohormesis, so activating mitochondrial turnover and renewal, but they could also disable mitochondrial support for viral replication, and perhaps, enhance apoptosis. However, this has to be balanced with the possibility that they could cause too much damage, and potentially, worsen the situation.

One group of drugs that has been investigated as a possible treatment for SARs-CoV-2 are the antimalarial aminoquinolones, which have been investigated for decades as immunomodulators and anti-virals. Their basic mode of action involves proton capture and deacidification of the lysosomal/endosomal compartment, which interferes with viral replication, autophagy and inflammatory pathways, but they also affect the plasma membrane, MAP kinases, calcium signalling, as well as DNA [185]. They can also modulate mitochondrial function [186–188] and have been shown to have anti-oxidant activity [189]. Paradoxically, they can also induce oxidative stress, which has raised concerns about their use in COVID-19 treatment due to the hypoxia associated with the acute respiratory distress syndrome (ARDS); one suggested mechanism is increased ROS generated by mitochondria – as well as possible direct effects on mitochondria [190]. A meta-analysis has shown that hydroxychloroquine used in treatment of COVID-19 resulted in a 2.5 times greater mortality compared to control groups, whereas its use was associated with a 1.2 times improvement in patients with mild to moderate symptoms compared to a control group [191]. A pharmacovigilance study also found that the use of hydrochloroquine/chloroquine for treatment of COVID19 was associated with higher rates of cardiovascular side effects [192]. Interestingly, in another study, although hydroxychloroquine and chloroquine were not associated with any significant effect on mortality, hydroxychloroquine, but not chloroquine, was associated with a significant reduction in transfer to the intensive care unit of patients admitted to hospital [193]. Critically, a recent review of the literature show these molecules to have biphasic/hormetic effects in multiple models, for instance, they can both stimulate or inhibit cancer cell and virus growth depending on dose [194]. This not only highlights the role of dose, but also, potentially, the induction of oxidative stress and the patient’s underlying health, and whether or not these compounds enhance risk or benefit.

Another very old anti-inflammatory drug, colchicine, is also being investigated for efficacy in COVID-19 patients, as it seems to inhibit the NLRP3 inflammasome, perhaps by suppressing the transport of mitochondria [195]. Interestingly, SARs-CoV-2 does seem to modulate many proteins related to the cytoskeleton, and can induce filopodial protusions [94]. Colchicine is often used to study autophagy, as it depolarises microtubules, so inhibiting the process. It has now been shown that it can result in impairments in skeletal mitochondrial function, increasing ROS, and in older animals, this can result in insulin resistance [196]. This all suggests that many of these drugs, especially those that might affect mitochondrial function, either directly, or indirectly, and could have an age-dependent effect - especially those that might affect autophagy.

An important class of drugs are the MAPK inhibitors, many of which have been developed as anti-cancer agents. As indicated previously, MAPKs can modulate mitochondrial function. They have been proposed as potential treatments as the virus seems to upregulate p38 activity and inhibit counter-regulatory pathways. Although this may well help in viral replication, it can also result in excessive inflammation in some patients [197, 198]. Interestingly, vemurafenib, a MAPK inhibitor, has been shown to inhibit dynamin-related protein 1 (DRP1) phosphorylation, reversing excessive mitochondrial fission in melanoma cells and resulting in hyper-fusion and enhancing oxidative phosphorylation and reversal of aerobic glycolysis [199]. This again highlights the parallels between cancer and viral infection in the sense that both induce extensive metabolic reprogramming and manipulation of the cell cycle, often towards aerobic glycolysis with modulation of mitochondrial function, as well as attenuating/modifying immune responses.

Data also suggest that cyclophilin inhibitors, such as cyclosporine A, which apart from being immune-suppressants, could also inhibit the replication of related corona viruses. Data has already shown that some transplant patients receiving immunosuppressants seemed to have some protection against the virus, although these were observational studies and other factors, such as good hygiene, could be important. But in vitro data does hint at efficacy, especially on other corona viruses, such as Middle East respiratory syndrome coronavirus (MERS), as does evidence around the importance of the cyclophilins in aiding viral replication. The mode of action is thought to involve inhibition of the calcineurin and suppression of the nuclear factor of activated T cells (NFAT) (reviewed in [200]). In mice infected with MERS-CoV, it seems that cyclosporine induces a robust interferon gamma response, which is associated with inhibition of viral replication and release [201]. MAVs are a key component of resistance to viruses, and can activate both interferon and NF-kB pathways, putting mitochondria centre stage in viral defence [202]; data indicate that immunophilins are regulators of MAVs [203, 204]. Given the importance of cyclophilin D, a well-known target of cyclosporine that modulates mitochondrial permeability transition [205], it would be interesting to speculate that apart from the well described immunophilin targets of compounds like tacrolimus and cyclosporine, a role for modulation of mitochondria could not be ruled out. In this light, the effectiveness of the pan-cyclophilin inhibitor, Alisporivir, which does not have immunosuppressive effects, is potentially interesting as it has high potency against SARS-CoV-2. It has been suggested that its ability to inhibit cyclophilin D, and thus control mitochondrial permeability, maybe of importance in preventing lung damage [206].

Finally, some very promising preliminary data from the RECOVERY trial suggests that low dose dexamethasone could help prevent death of up to 30% of ventilated patients [207]. On the 18th September 2020, the European Medicines agency endorsed the use of dexamethasone in COVID-19 patients on oxygen or mechanical ventilation (EMA/483739/2020). The most well-known effect of glucocorticoids is to suppress inflammation, largely through the glucorticoid receptor (GR), but with chronic use they do have side effects, as they are catabolic [208]. The key point here is that glucocorticoids are generally induced by stress, and in the short term, are highly protective. It is thus relevant that GRs also transfer to the mitochondrion and control mitochondrial gene transcription, and have biphasic actions [209]. It is thus relevant that dexamethasone has been shown to both induce mitochondrial uncoupling and increase oxidative phosphorylation [210], but also cause mitochondrial dysfunction [211]. This is hardly surprising as mitochondria are central to both steroid biosynthesis and action, and thus, stress management [174]. Although it might be surmised that the predominant effect in the RECOVERY trial is through direct suppression of inflammatory pathways, it is not impossible that effects on mitochondria could not be ruled out.

### Anti-oxidants and natural products

A further approach that has been suggested is suppression of oxidative stress by using compounds that are anti-oxidants. Direct anti-oxidants, for example N-acetyl cysteine, which, although it has shown some efficacy, has met with limited success due to dose issues [212]. However vitamin C, which is now known to concentrate in mitochondria and act as a ROS scavenger [213], could be useful. A retrospective analysis of data has suggested that vitamin C can both reduce the time in the intensive care unit and the time on ventilators, particularly for very ill patients [214, 215]. It is now being suggested that it is used in combination with quercetin, which also seems to have efficacy in viral infections [216]: quercetin is a natural product that has anti-oxidant properties and concentrates in mitochondria and can induce mitochondrial biogenesis [217, 218].

Another important principle is that many plant compounds seem to have anti-viral properties, as well as anti-cancer properties, and modulate calcium signalling and mitochondrial function – with common targets, such as VDAC. Plants suffer both from viral infection and cancer, so it could be that there is some cross over in function from plants to animals [219]. As viruses seem to hijack their host’s cellular machinery, including mitochondrial function, then partially inhibiting mitochondrial function could be an evolved strategy to defeat the virus, especially if it induces apoptosis and/or upregulates mitophagy and mitochondrial renewal and anti-oxidant systems. A good example of this is perhaps salicylic acid, which is a major plant defence signalling compound [220] and modulates mitochondrial function, both inhibiting the electron transport chain and acting as an uncoupling agent [221, 222], as well as regulating VDAC expression [223]. Some plant viruses produce proteins that can inhibit the oxidative burst and salicyclic-acid dependent autophagy [224]. There is thus, potentially, useful insight provided by the observation that some medicines that are derived from plant (or other organism) defence compounds, also appear to have some benefit in human viral infections. Indeed, Gurbel and colleagues have suggested that aspirin could be used against SARS-CoV-2, for instance, by reducing its activation of NF-κB [225].

There is also interest in the potential for compounds such as cannabidiol (CBD) in helping COVID-19 patients, as the cannabinoids do seem to have some anti-viral activity, and are anti-oxidant and anti-inflammatory [226]. CBD, amongst its many identified targets [227], does seem to directly modulate mitochondrial function, for instance, it has been shown to bind to VDAC1 and inhibit the electron transport chain [228, 229]. There is also evidence that it can inhibit inflammasome activation [230].

One key mechanism is that many plant compounds activate Nrf2 and are thus hormetic [231]. Furthermore, as many manufactured drugs were developed from defence compounds found in plants and other organisms, this principle could be extended to include them. Another example of this could be the statins, which also inhibit mitochondrial function [232–234], and one study has indeed shown they can reduce mortality of COVID-19 patients [235].

Another ubiquitous antioxidant molecule, melatonin, which also protects mitochondria [236], is also being investigated as an adjuvant to protect against a cytokine storm in SARs-CoV-2 infection [237]. Interestingly, it has been shown to reverse the Warburg effect in immune cells, potentially having an anti-inflammatory effect, and providing a justification for its use in COVID-19 patients [92]. Likewise, glutathione is also showing promise, in particular, as it seems to help redress the age related Th1/Th2 imbalance [160]. Of potential relevance is the observation that a modified vitamin E derivative that concentrates in mitochondria has shown benefits in a model of cardiac inflammation induced by sepsis. It seems to do this by suppressing mitochondrial DNA damage and its subsequent release [238]; mtDNA is a potent activator of the inflammatory system [239].

Also of interest here is Vitamin D, which has been suggested as a potential adjuvant treatment for patients with the virus, as it may restore immune function. In particular, it may enhance anti-inflammatory cytokine production and so limit the possibility of a cytokine storm. Analyses do seem to show that it can have some benefit in people who have low levels of this vitamin [240]. Critically, it modulates mitochondrial function, having diverse affects depending on the tissue; it can stimulate muscle mitochondrial function [241], but may also enhance lipid storage and adipogenesis [242]. Interestingly, it has been suggested that COVID-19 morbidity increases with northerly latitude, suggesting a link with ultraviolet light and vitamin D [243]. Vitamin B3 has also been shown to have some protective effects in mitochondrial myopathy models [244], and has been suggested that it could help prevent lung injury in COVID-19 patients [245].

Finally, artificial mitochondrial anti-oxidant molecules, such as MitoQ and SKQ1 could also provide benefit [246]. There are also compounds like Luminol derivatives, which only become ROS scavengers in areas of high oxidative stress and are showing some promise in modulating redox-driven inflammation [247–249]. However, unlike MitoQ and SKQ1, it is likely that Luminol-like compounds act outside the mitochondrion [250].

### Vaccination

The age related decline in immune function is well described, although not well understood, and affects virtually all components and has a big impact on the success of vaccination – which has led to a constant drive to improve vaccines for the older generation, in particular against influenza [251]. It is, however, recognised to be modifiable by many factors, ranging from exercise, to stress, and chronic infections [252]. Critical in these responses is metabolic flexibility, for instance, the ability to switch between oxidative phosphorylation and glycolysis, and how this effects different sub-populations of cells and the pro-inflammatory/anti-inflammatory balance. For instance, aged B-cells lose oxidative phosphorylation capacity, and rely more on glycolysis and generate more ROS. They also infiltrate adipose tissue, heightening inflammation in a process involving the NLRP3 inflammasome. In relation to the B-cell response, which is key purpose of vaccination, it seems that obesity, and the metabolic syndrome, accelerates immunosenescence and reduces the ability to produce antibodies [253].

A primary research area is on the development of vaccines for the elderly against influenza; on average, over the age of 65 years, the efficacy drops off rapidly. To study this, immunosenescence related markers in the blood have been correlated with outcome. Interestingly, T-cell responses have been found to be a stronger correlates of protection than antibodies. Although the biology is immensely complex, and may require a system level “vaccinomics” approach, dysregulated metabolism is clearly part of the problem – and it has been suggested that treatments to correct dysregulated metabolic or other physiological processes may be required before administration of vaccines [254].

It would therefore seem that in order to improve the efficacy of vaccines, it is either necessary to tailor the vaccine to the particular immunosenescence profile a patient shows, or, perhaps to reduce their epigenetic age by ensuring they live a healthy lifestyle and so enhance their immune system.

## Implications of SARs-CoV-2 modulation of mitochondrial function

There are a number of factors to consider from this. If, as seems to be the case, this virus is modulating mitochondrial function, and thus, mitochondrial health is important, there are a number of intriguing possibilities.

### Does mitochondrial function explain why morbidity may be greater among men than women?

There are obviously confounding behavioural factors, but statistically, men seem to have higher rates of mortality than women when infected with the coronavirus [255, 256]. Mitochondria in females may be more robust, which could explain why females tend to live longer than males [257].

### Pollution, mitochondria and severity

Would pollution lower resistance to the virus? Nitrogen dioxide is oxidative and can induce pulmonary inflammation and reduce function [258], oxidise mitochondrial cytochrome C [259], while acute inhalation can cause mitochondrial dysfunction in the brain [260]. Data from the United Kingdom is now suggesting that high levels of pollution are linked to increased COVID-19 lethality [261]. Linked to this is the very disturbing evidence that iron-rich nanoparticles, largely derived from motor vehicles, are now being found in cardiac mitochondria in the very young, and are causing oxidative stress [262].

### The renin-angiotensin-aldosterone system (RAAS) and mitochondrial function

The coronavirus binds to ACE2 [263, 264] and mitochondria have their own angiotensin system [85]. ACE2 cleaves angiotensin II to produce anti-inflammatory molecules and protects mitochondria [84, 86]. This suggests ACE1/2 polymorphisms will be a factor in reaction to the virus [265]. ARBs, ACEi and statins may enhance ACE2 activity. Their role in treatment is thus debated [266, 267].

### Hypoxic-ischaemic reperfusion injury and oxygen

During hypoxia, mitochondrial function is inhibited, but then becomes a source of ROS during reperfusion. Could damaged mitochondria in the lung and/or heart lead to an exacerbation of symptoms if too much oxygen is given to a patient? This is clearly a difficult clinical conundrum, but does suggest that supplementary oxygen should only be used where absolutely necessary. Compounds such as melatonin, CBD and curcumin have shown some protective effects ischaemic-reperfusion models [268–270] – curcumin is an uncoupling agent [271]. CBD modulates mitochondria [228]. Key in this is emerging data that hypoxic preconditioning requires a drop in the mitochondrial proton motive force [272]. Management of ETC uncoupling is thus vital for life to control oxidative stress [273]. PPARs may play a key role in controlling uncoupling [274]. Furthermore there is much evidence that anaesthetics can modulate mitochondrial function and could play a role in both pre- and post-ischaemia protection, and some can act as uncoupling agents [275–277].

### Phytochemical viral protease inhibitors and mitochondria

Two recent structure-docking studies have indicated that several phytochemicals could inhibit the SARs-CoV-2 protease [278, 279]. As many phytochemicals can also modulate mitochondrial function [280], and primarily evolved to protect the plant, it could be surmised that they are multi-functional and modulate multiple pathways to achieve this.

### Long term effects – “long covid”

It is becoming clear that following recovery from the primary infection with SARS-CoV-2, many people are suffering from long term effects, such as fatigue and mental health problems, as well as more obvious lung problems. This has resulted in the formation of a national UK consortium and the launch of the PHOSP-COVID study to investigate the long terms effects on health of this virus (see https://www.phosp.org/) [281]. One possible consequence of viral infection could be longer term mitochondrial dysfunction, which could lead to a variety of symptoms. Mitochondrial function, and their relationship to immunity, is again becoming a focus for research in the chronic fatigue syndrome, which is still not completely understood [282]. This has been further supported by evidence of mitochondrial dysfunction in PBMCs of people with chronic fatigue syndrome [283].

## Can we test the hypothesis that mitochondrial health = immune health and enhanced resistance to the virus?

In terms of testing and/or looking for evidence that mitochondria could help explain some of the pathophysiology of this virus, there are several potential ways to look for this relationship, ranging from laboratory based to population studies.

### Direct evidence that SARs-CoV-2 modulates mitochondrial function

This work could be carried out in vitro with cultured cells and/or isolated mitochondria prepared from control and infected individuals. In particular, using imaging to look for co-localisation in “virus factories”.

### Lifestyle and mitochondrial function

It could be predicted that those populations exhibiting the lowest levels of optimal health and the highest levels of the metabolic syndrome and “diabesity”, will show the highest susceptibility. For example, it might be revealing to map the case fatality rate to the latest trends in obesity/ diabetes after the necessary confounders are taken into account [284]. In support of this, the emerging data from New York in relation to SARS-CoV-2 infection is that obesity is strongly correlated with critical illness [9]. In contrast, would those populations showing the highest fitness levels and functioning be more resistant? For instance, would measured VO_2_ max show an inverse relationship with morbidity?

### Inherited mitochondrial dysfunction

Individuals with known mitochondrial dysfunction are well known to show abnormal susceptibility to infections [25]. Is there a link between mitochondrial haplotype and resistance? There is certainly evidence for different mtDNA haplotypes amongst different populations [285]. Although there is an emerging disparity in morbidity between Black people and other minorities in the USA, it is thought it may be more to do with socio-economic imbalances and higher rates of lifestyle induced co-morbidities [286].

### Markers of mitochondrial health in the blood

Blood-derived mitochondrial markers of reduced function may correlate with disease severity before, during and after infection.

### Epigenetic age and mitochondrial function

Data now show it is possible to determine someone’s epigenetic age and compare it with their chronological age. There is a close correlation with this ratio and co-existing morbidity [287]. Thus, would blood-derived epigenetic markers of metabolic age correlate with disease severity before, during and after infection?

## Conclusion

The main conclusion from this review is that as immune function is dependent on mitochondrial function, and although this does decline with age, the rate it does so can be modified by lifestyle. This is perhaps best highlighted by the link between ageing, mitochondrial function and the metabolic syndrome. This implies that both resistance to the virus, and the effectiveness of a vaccine, will be linked to the mitochondrial health of the individual. Furthermore, as evidence indicates that many viruses, which most likely include SARs-CoV-2, modulate bioenergetics and redox in both the immune system and other cells they infect to enhance their own replication, they could potentially induce excessive stress in these systems if their mitochondria are already sub-optimally functional. This would suggest that in patients experiencing severer symptoms, mitochondrial support could be a strategy, which could take many forms, both direct (e.g., mitochondrial anti-oxidants), or indirect (anti-inflammatories, inhibitors of viral replication etc.). This viewpoint becomes apparent when one considers that mitochondria are a central nexus and have many functions, ranging from supplying energy, anabolites for new growth, controlling intracellular redox, calcium signalling, detection of viruses and activation of anti-viral mechanisms, as well as ultimately controlling the life and death of the cell.

## Data Availability

Not applicable.

## Abbreviations

ACE2: Angiotensin-converting enzyme 2
AMPK: Adenosine monophosphate kinase
ARB: Angiotensin receptor blocker
AT2: Pulmonary alveolar type 2 progenitor cells
CBD: Cannabidiol
COX: Cyclooxygenase
CRP: C-reactive peptide
DPP4: Dipeptidyl peptidase 4
DNAmAge: Chronological age through DNA methylation
DRP-1: Dynamin-like protein 1
ERGIC: Endoplasmic reticulum-Golgi intermediate compartment
EV: Extracellular vesicle
GR: Glucorticoid receptor
HIF-1α: Hypoxia-inducible factor-1α
IFN1: Type 1 interferon
IL-1: Interleukin-1
IL-6: Interleukin-6
IL-β: Interleukin beta
IR: Ischaemia/reperfusion
MAPK: P38 mitogen activated protein kinase
MAVs: Mitochondrial anti-viral signalling proteins
MERS: Middle East respiratory syndrome coronavirus
mtDNAcn: Mitochondrial DNA copy number
mTOR: Mammalian target of rapamycin
Nrp-1: Neuropilin-1
NF-κB: Nuclear factor kappa B
NFAT: Nuclear factor of activated T cells
NLRP1/3: Nod-like receptor pyrin family domain containing 1/3
NRF2: Nuclear factor erythroid 2-like 2
NSAID: Non-steroidal anti-inflammatory drug
ORF: Open reading frame
PPAR: Peroxisomal proliferating activated receptor
PGC1α: Peroxisomal proliferator-activated receptor γ coactivator 1α
RCR: Respiratory control ratio
R-ORB: Redox-optimised ROS balance
ROS: Reactive oxygen species
SARS-CoV-2: Severe acute respiratory syndrome coronavirus 2
T2D: Type 2 diabetes
Th1: T helper 1
TNFα: Tumour necrosis factor alpha
UCP2: Uncoupling protein 2
VAT: Visceral adipose tissue
VDAC1: Voltage dependent anion channel 1
VEGF: Vascular endothelial growth factor
